# The genome sequence of the Acer Sober, *Anarsia innoxiella *(Gregersen & Karsholt, 2017)

**DOI:** 10.12688/wellcomeopenres.19514.1

**Published:** 2023-08-18

**Authors:** Douglas Boyes, Clare Boyes, University of Oxford and Wytham Woods Genome Acquisition Lab, Darwin Tree of Life Barcoding collective, Wellcome Sanger Institute Tree of Life programme, Wellcome Sanger Institute Scientific Operations: DNA Pipelines collective, Tree of Life Core Informatics collective, Darwin Tree of Life Consortium

**Affiliations:** 1 UK Centre for Ecology & Hydrology, Wallingford, England, UK; 2 Independent researcher, Welshpool, Wales, UK

**Keywords:** Anarsia innoxiella, Acer Sober, genome sequence, chromosomal, Lepidoptera

## Abstract

We present a genome assembly from an individual female *Anarsia innoxiella* (the Acer Sober; Arthropoda; Insecta; Lepidoptera; Gelechiidae). The genome sequence is 302.9 megabases in span. Most of the assembly is scaffolded into 31 chromosomal pseudomolecules, including the Z and W sex chromosomes. The mitochondrial genome has also been assembled and is 15.25 kilobases in length.

## Species taxonomy

Eukaryota; Metazoa; Eumetazoa; Bilateria; Protostomia; Ecdysozoa; Panarthropoda; Arthropoda; Mandibulata; Pancrustacea; Hexapoda; Insecta; Dicondylia; Pterygota; Neoptera; Endopterygota; Amphiesmenoptera; Lepidoptera; Glossata; Neolepidoptera; Heteroneura; Ditrysia; Gelechioidea; Gelechiidae; Anacampsinae; *Anarsia*; *Anarsia innoxiella* ( Gregersen & Karsholt, 2017) (NCBI:txid2566270).

## Background

*Anarsia innoxiella* (Acer Sober) is a leaf-mining micro-moth in the family Gelechiidae which was described as new to science in 2017. The moth was found to be a distinct species from the very similar *A. lineatella*, which is a serious pest of fruit trees ( *Prunus* spp). However, the larvae of *A. innoxiella* feed on *Acer* species (Sapindaceae), particularly field maple ( *Acer campestre*). This difference led to the suspicion that *A. innoxiella* was a separate species; and this was confirmed by DNA barcoding. Taxonomic work found subtle morphological differences between the two species ( Gregersen & Karsholt, 2017).

This small adult moth (forewing length 6–8 mm) has light and dark grey mottled forewings with black longitudinal streaks, and an especially prominent streak in the middle of the wing. There is some variation in appearance with some adults appearing light and variegated; and others appearing darker, and more closely resembling *A. lineatella*. In males, the species can be confirmed by genital dissection ( Palmer, 2017).

As the species is newly described, information about its distribution worldwide is not complete, but it appears to be widespread in central Europe and southern Scandinavia, and can be locally common ( GBIF Secretariat, 2023). In the UK, critical re-examination of previously determined specimens of *A. lineatella* has found the earliest confirmed record of *A. innoxiella* to be in 1991 from west Sussex. It has since been verified from many southern counties and it is believed that most, but not all, of the previous records of *A. lineatella* are actually *A. innoxiella*. Since 1991, it has occurred annually and these records suggest that the moth is single-brooded, flying between late June and early August ( Palmer, 2017).

A genome sequence from *A. innoxiella* will be useful for further research into this cryptic group of moths. The genome of *A. innoxiella* was sequenced as part of the Darwin Tree of Life Project, a collaborative effort to sequence all the named eukaryotic species in the Atlantic Archipelago of Britain and Ireland. Here we present a chromosomally complete genome sequence for *A. innoxiella* based on a female specimen from Wytham Woods, Oxfordshire, UK.

## Genome sequence report

**Figure 1.**
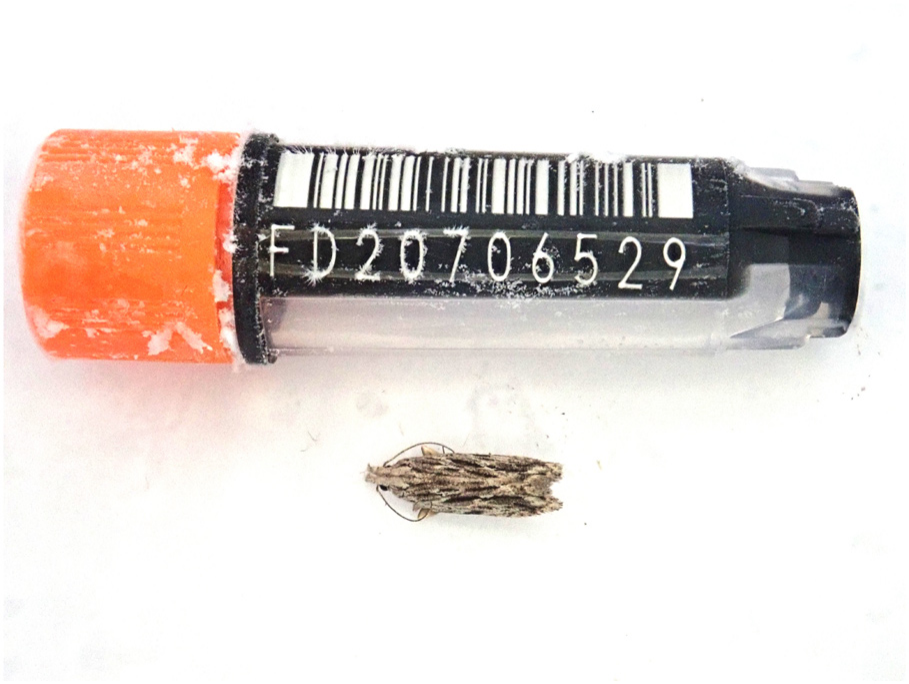
Photograph of the *Anarsia innoxiella* (ilAnaInnx2) specimen used for genome sequencing.

The genome was sequenced from one female *Anarsia innoxiella* ( Figure 1) collected from Wytham Woods, Oxfordshire, UK (51.77, –1.31). A total of 78-fold coverage in Pacific Biosciences single-molecule HiFi long was generated. Primary assembly contigs were scaffolded with chromosome conformation Hi-C data. Manual assembly curation corrected 23 missing joins or mis-joins and removed two haplotypic duplications, reducing the scaffold number by 20%, and increasing the scaffold N50 by 1.3%.

**Figure 2.**
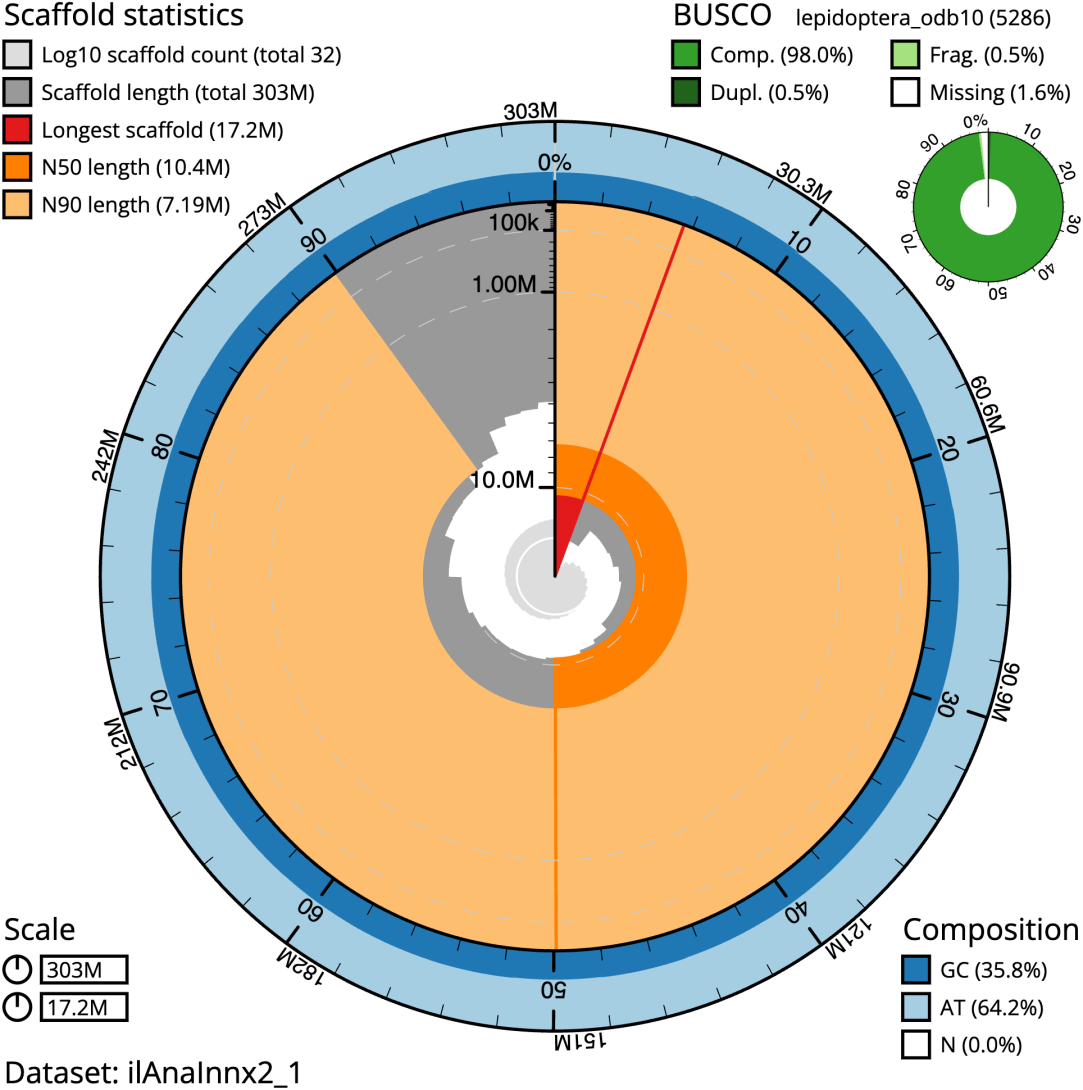
Genome assembly of *Anarsia innoxiella*, ilAnaInnx2.1: metrics. The BlobToolKit Snailplot shows N50 metrics and BUSCO gene completeness. The main plot is divided into 1,000 size-ordered bins around the circumference with each bin representing 0.1% of the 302,928,861 bp assembly. The distribution of scaffold lengths is shown in dark grey with the plot radius scaled to the longest scaffold present in the assembly (17,173,194 bp, shown in red). . Orange and pale-orange arcs show the N50 and N90 scaffold lengths (10,425,486 and 7,192,583 bp), respectively. The pale grey spiral shows the cumulative scaffold count on a log scale with white scale lines showing successive orders of magnitude. The blue and pale-blue area around the outside of the plot shows the distribution of GC, AT and N percentages in the same bins as the inner plot. A summary of complete, fragmented, duplicated and missing BUSCO genes in the lepidoptera_odb10 set is shown in the top right. An interactive version of this figure is available at https://blobtoolkit.genomehubs.org/view/ilAnaInnx2_1/dataset/ilAnaInnx2_1/snail.

The final assembly has a total length of 302.9 Mb in 31 sequence scaffolds with a scaffold N50 of 10.4 Mb ( Table 1). Most (99.99%) of the assembly sequence was assigned to 31 chromosomal-level scaffolds, representing 29 autosomes and the W and Z sex chromosomes. Chromosome-scale scaffolds confirmed by the Hi-C data are named in order of size ( Figure 2– Figure 5; Table 2). While not fully phased, the assembly deposited is of one haplotype. Contigs corresponding to the second haplotype have also been deposited. The mitochondrial genome was also assembled and can be found as a contig within the multifasta file of the genome submission.

**Table 1.** Genome data for *Anarsia innoxiella*, ilAnaInnx2.1.

Project accession data		
Assembly identifier	ilAnaInnx2.1	
Species	*Anarsia innoxiella*	
Specimen	ilAnaInnx2	
NCBI taxonomy ID	2566270	
BioProject	PRJEB56247	
BioSample ID	SAMEA10978939	
Isolate information	ilAnaInnx2, female: whole organism (DNA sequencing) ilAnaInnx1, whole organism (Hi-C scaffolding)	
Assembly metrics *		*Benchmark*
Consensus quality (QV)	64.4	*≥ 50*
*k*-mer completeness	100%	*≥ 95%*
BUSCO **	C:98.0%[S:97.4%,D:0.5%], F:0.5%,M:1.6%,n:5,286	*C ≥ 95%*
Percentage of assembly mapped to chromosomes	99.99%	*≥ 95%*
Sex chromosomes	Z and W chromosomes	*localised homologous pairs*
Organelles	Mitochondrial genome assembled	*complete single alleles*
Raw data accessions		
PacificBiosciences SEQUEL II	ERR10355966	
Hi-C Illumina	ERR10297862	
Genome assembly		
Assembly accession	GCA_947563765.1	
*Accession of alternate haplotype*	GCA_947563745.1	
Span (Mb)	302.9	
Number of contigs	90	
Contig N50 length (Mb)	5.8	
Number of scaffolds	32	
Scaffold N50 length (Mb)	10.4	
Longest scaffold (Mb)	17.2	

* Assembly metric benchmarks are adapted from column VGP-2020 of “Table 1: Proposed standards and metrics for defining genome assembly quality” from ( Rhie *et al.*, 2021). ** BUSCO scores based on the lepidoptera_odb10 BUSCO set using v5.3.2. C = complete [S = single copy, D = duplicated], F = fragmented, M = missing, n = number of orthologues in comparison. A full set of BUSCO scores is available at https://blobtoolkit.genomehubs.org/view/ilAnaInnx2_1/dataset/ilAnaInnx2_1/busco.

**Figure 3.**
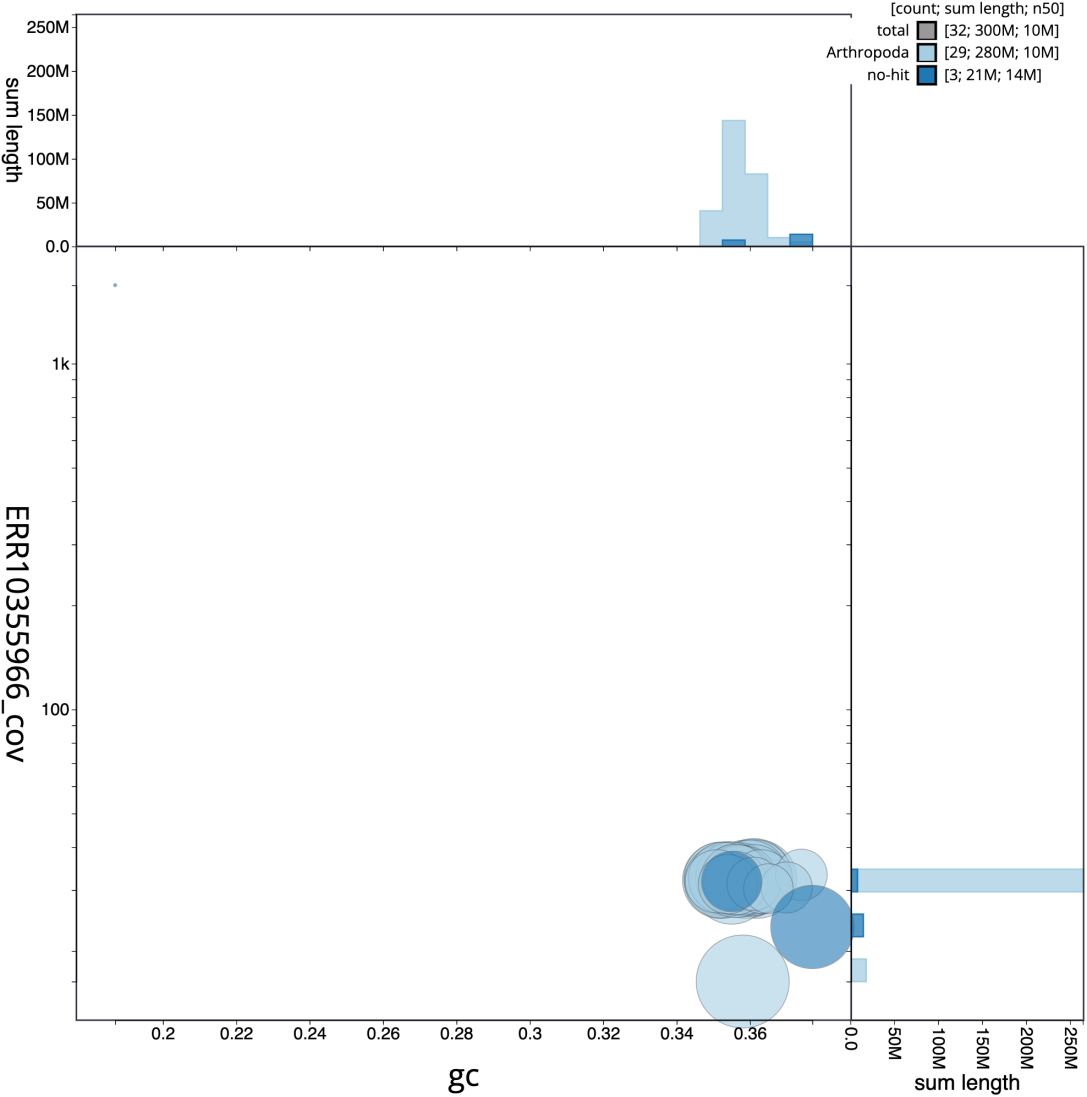
Genome assembly of *Anarsia innoxiella*, ilAnaInnx2.1: BlobToolKit GC-coverage plot. Scaffolds are coloured by phylum. Circles are sized in proportion to scaffold length. Histograms show the distribution of scaffold length sum along each axis. An interactive version of this figure is available at https://blobtoolkit.genomehubs.org/view/ilAnaInnx2_1/dataset/ilAnaInnx2_1/blob.

**Figure 4.**
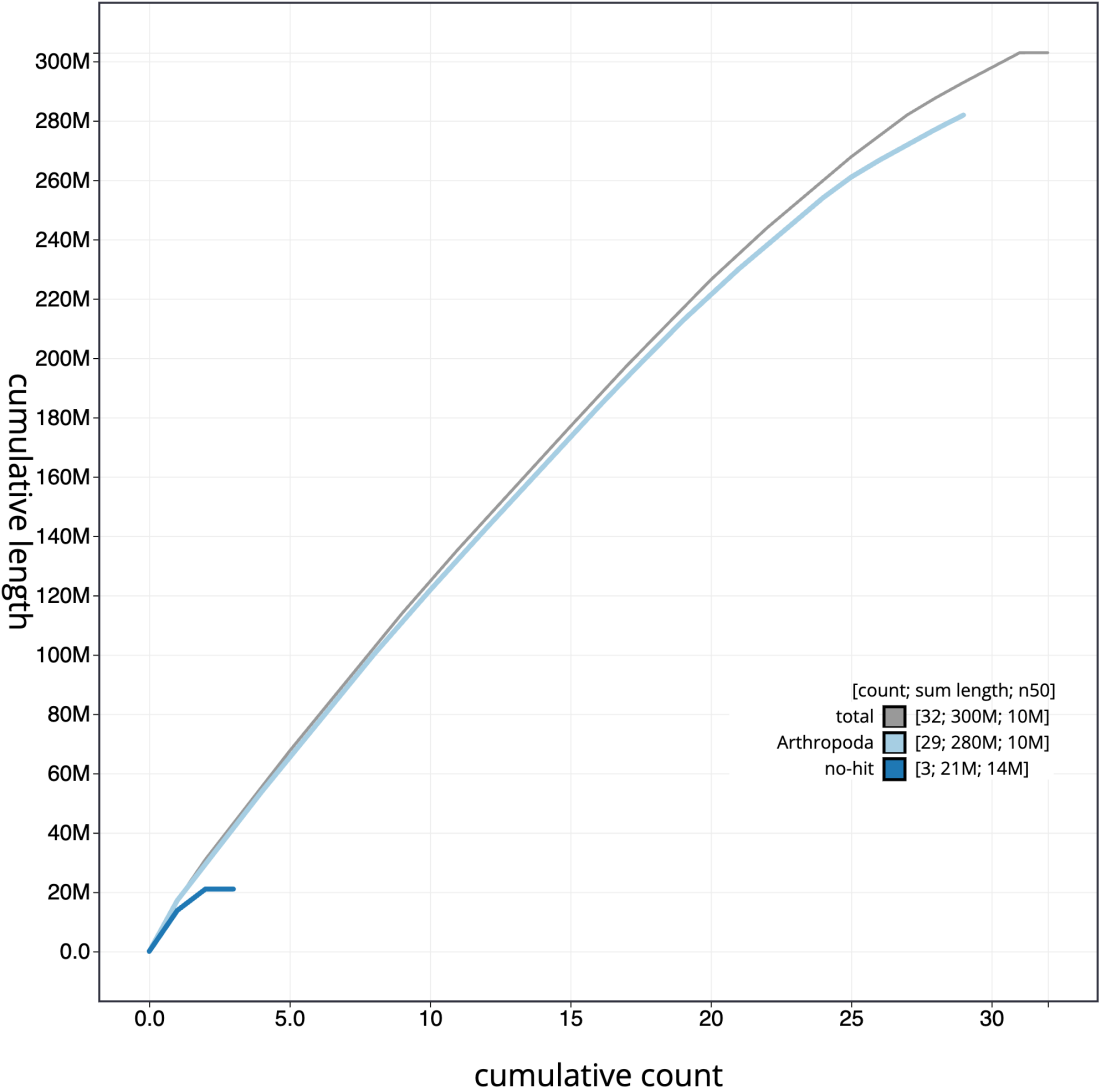
Genome assembly of *Anarsia innoxiella*, ilAnaInnx2.1: BlobToolKit cumulative sequence plot. The grey line shows cumulative length for all scaffolds. Coloured lines show cumulative lengths of scaffolds assigned to each phylum using the buscogenes taxrule. An interactive version of this figure is available at https://blobtoolkit.genomehubs.org/view/ilAnaInnx2_1/dataset/ilAnaInnx2_1/cumulative.

**Figure 5.**
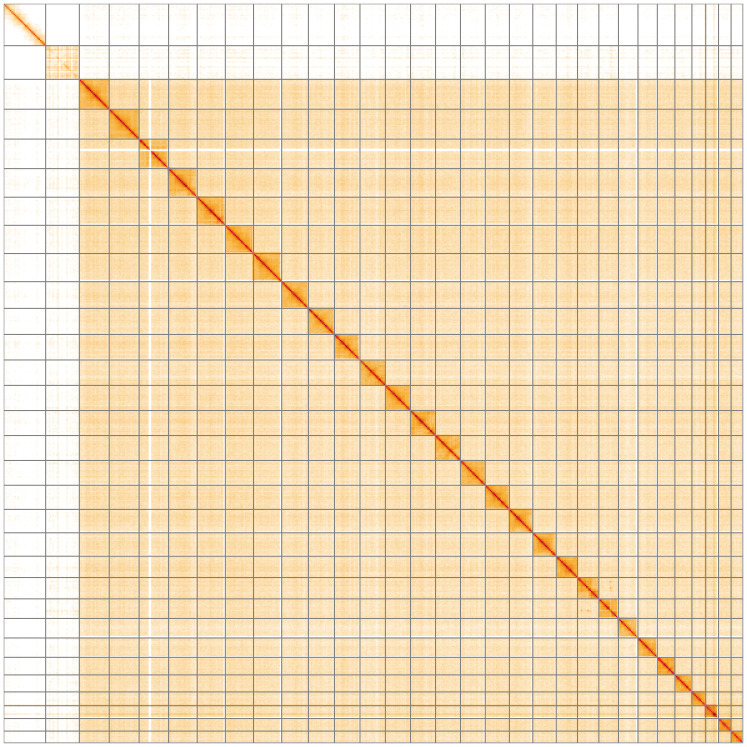
Genome assembly of *Anarsia innoxiella*, ilAnaInnx2.1: Hi-C contact map of the ilAnaInnx2.1 assembly, visualised using HiGlass. Chromosomes are shown in order of size from left to right and top to bottom. An interactive version of this figure may be viewed at https://genome-note-higlass.tol.sanger.ac.uk/l/?d=Vjcc-TrMQ5ua30J26JKCBg.

**Table 2.** Chromosomal pseudomolecules in the genome assembly of *Anarsia innoxiella*, ilAnaInnx2.

INSDC accession	Chromosome	Length (Mb)	GC%
OX387392.1	1	12.24	36.0
OX387393.1	2	12.24	36.0
OX387394.1	3	12.11	35.5
OX387395.1	4	11.72	36.0
OX387396.1	5	11.54	36.0
OX387397.1	6	11.53	36.0
OX387398.1	7	11.53	35.0
OX387399.1	8	10.97	35.5
OX387400.1	9	10.69	35.0
OX387401.1	10	10.44	35.5
OX387402.1	11	10.43	35.0
OX387403.1	12	10.29	35.5
OX387404.1	13	10.26	35.5
OX387405.1	14	10.21	35.5
OX387406.1	15	10.18	35.5
OX387407.1	16	9.87	35.5
OX387408.1	17	9.59	36.0
OX387409.1	18	9.59	36.0
OX387410.1	19	8.74	35.5
OX387411.1	20	8.73	35.5
OX387412.1	21	8.04	36.5
OX387413.1	22	7.99	35.5
OX387414.1	23	7.95	35.0
OX387415.1	24	7.19	35.5
OX387416.1	25	6.96	35.5
OX387417.1	26	5.67	36.0
OX387418.1	27	5.25	37.5
OX387419.1	28	5.16	37.0
OX387420.1	29	4.85	36.5
OX387391.1	W	13.8	37.5
OX387390.1	Z	17.17	36.0
OX387421.1	MT	0.02	19.0

The estimated Quality Value (QV) of the final assembly is 64.4 with *k*-mer completeness of 100%, and the assembly has a BUSCO v5.3.2 completeness of 98.0%% (single =97.4%, duplicated = 0.5%), using the lepidoptera_odb10 reference set ( *n* =5,286).

Metadata for specimens, spectral estimates, sequencing runs, contaminants and pre-curation assembly statistics can be found at https://links.tol.sanger.ac.uk/species/2566270.

## Methods

### Sample acquisition and nucleic acid extraction

A female *Anarsia innoxiella* (ilAnaInnx2) was collected from Wytham Woods, Oxfordshire (biological vice-county Berkshire), UK (latitude 51.77, longitude –1.31) on 2021-07-17. The specimen was taken from woodland habitat using a light trap. This specimen was used for DNA sequencing. A second specimen (ilAnaInnx1) was collected from Wytham Woods (latitude 51.77, longitude –1.31) on 2020-08-01. This specimen was used for Hi-C scaffolding. Both specimens were collected and identified by Douglas Boyes (University of Oxford) and were preserved on dry ice.

The sample was prepared for DNA extraction extracted at the Tree of Life laboratory, Wellcome Sanger Institute (WSI). The ilAnaInnx2 sample was weighed and dissected on dry ice with tissue set aside for Hi-C sequencing. Whole organism tissue was disrupted using a Nippi Powermasher fitted with a BioMasher pestle. DNA was extracted from whole organism tissue of ilAnaInnx2 at the Wellcome Sanger Institute (WSI) Scientific Operations core using the Qiagen MagAttract HMW DNA kit, according to the manufacturer’s instructions.

### Sequencing

Pacific Biosciences HiFi circular consensus DNA sequencing libraries were constructed according to the manufacturers’ instructions. DNA sequencing was performed by the Scientific Operations core at the WSI on Pacific Biosciences SEQUEL II (HiFi) instrument. Hi-C data were also generated from whole organism tissue of ilAnaInnx1 using the Arima2 kit and sequenced on the Illumina NovaSeq 6000 instrument.

### Genome assembly, curation and evaluation

Assembly was carried out with Hifiasm ( Cheng *et al.*, 2021) and haplotypic duplication was identified and removed with purge_dups ( Guan *et al.*, 2020). The assembly was then scaffolded with Hi-C data ( Rao *et al.*, 2014) using YaHS ( Zhou *et al.*, 2023). The assembly was checked for contamination as described previously ( Howe *et al.*, 2021). Manual curation was performed using HiGlass ( Kerpedjiev *et al.*, 2018) and Pretext ( Harry, 2022). The mitochondrial genome was assembled using MitoHiFi ( Uliano-Silva *et al.*, 2022), which runs MitoFinder ( Allio *et al.*, 2020) or MITOS ( Bernt *et al.*, 2013) and uses these annotations to select the final mitochondrial contig and to ensure the general quality of the sequence.

A Hi-C map for the final assembly was produced using bwa-mem2 ( Vasimuddin *et al.*, 2019) in the Cooler file format ( Abdennur & Mirny, 2020). To assess the assembly metrics, the *k*-mer completeness and QV consensus quality values were calculated in Merqury ( Rhie *et al.*, 2020). This work was done using Nextflow ( Di Tommaso *et al.*, 2017) DSL2 pipelines “sanger-tol/readmapping” ( Surana *et al.*, 2023a) and “sanger-tol/genomenote” ( Surana *et al.*, 2023b). The genome was analysed within the BlobToolKit environment ( Challis *et al.*, 2020) and BUSCO scores ( Manni *et al.*, 2021; Simão *et al.*, 2015) were calculated.

Table 3 contains a list of relevant software tool versions and sources.

**Table 3.** Software tools: versions and sources.

Software tool	Version	Source
BlobToolKit	4.1.3	https://github.com/blobtoolkit/blobtoolkit
BUSCO	5.3.2	https://gitlab.com/ezlab/busco
Hifiasm	0.16.1-r375	https://github.com/chhylp123/hifiasm
HiGlass	1.11.6	https://github.com/higlass/higlass
Merqury	MerquryFK	https://github.com/thegenemyers/MERQURY.FK
MitoHiFi	2	https://github.com/marcelauliano/MitoHiFi
PretextView	0.2	https://github.com/wtsi-hpag/PretextView
purge_dups	1.2.3	https://github.com/dfguan/purge_dups
sanger-tol/genomenote	v1.0	https://github.com/sanger-tol/genomenote
sanger-tol/readmapping	1.1.0	https://github.com/sanger-tol/readmapping/tree/1.1.0
YaHS	yahs-1.1.91eebc2	https://github.com/c-zhou/yahs

### Wellcome Sanger Institute – Legal and Governance

The materials that have contributed to this genome note have been supplied by a Darwin Tree of Life Partner.

The submission of materials by a Darwin Tree of Life Partner is subject to the **‘Darwin Tree of Life Project Sampling Code of Practice’**, which can be found in full on the Darwin Tree of Life website here. By agreeing with and signing up to the Sampling Code of Practice, the Darwin Tree of Life Partner agrees they will meet the legal and ethical requirements and standards set out within this document in respect of all samples acquired for, and supplied to, the Darwin Tree of Life Project.

Further, the Wellcome Sanger Institute employs a process whereby due diligence is carried out proportionate to the nature of the materials themselves, and the circumstances under which they have been/are to be collected and provided for use. The purpose of this is to address and mitigate any potential legal and/or ethical implications of receipt and use of the materials as part of the research project, and to ensure that in doing so we align with best practice wherever possible.

The overarching areas of consideration are:

- Ethical review of provenance and sourcing of the material
- Legality of collection, transfer and use (national and international)

Each transfer of samples is further undertaken according to a Research Collaboration Agreement or Material Transfer Agreement entered into by the Darwin Tree of Life Partner, Genome Research Limited (operating as the Wellcome Sanger Institute), and in some circumstances other Darwin Tree of Life collaborators.

## Data Availability

European Nucleotide Archive: Anarsia innoxiella (acer sober). Accession number PRJEB56247; https://identifiers.org/ena.embl/PRJEB56247. ( Wellcome Sanger Institute, 2022) The genome sequence is released openly for reuse. The *Anarsia innoxiella* genome sequencing initiative is part of the Darwin Tree of Life (DToL) project. All raw sequence data and the assembly have been deposited in INSDC databases. Raw data and assembly accession identifiers are reported in Table 1.

## Author information

Members of the University of Oxford and Wytham Woods Genome Acquisition Lab are listed here: https://doi.org/10.5281/zenodo.4789928.

Members of the Darwin Tree of Life Barcoding collective are listed here: https://doi.org/10.5281/zenodo.4893703.

Members of the Wellcome Sanger Institute Tree of Life programme are listed here: https://doi.org/10.5281/zenodo.4783585.

Members of Wellcome Sanger Institute Scientific Operations: DNA Pipelines collective are listed here: https://doi.org/10.5281/zenodo.4790455.

Members of the Tree of Life Core Informatics collective are listed here: https://doi.org/10.5281/zenodo.5013541.

Members of the Darwin Tree of Life Consortium are listed here: https://doi.org/10.5281/zenodo.4783558.
