# Supplementary material for: The genome sequence of the Acer Sober, Anarsia innoxiella (Gregersen & Karsholt, 2017)
Source: Wellcome Open Res. 2023 Aug 18;8:357. [Version 1] doi: 10.12688/wellcomeopenres.19514.1 (PMC11320181; doi:10.12688/wellcomeopenres.19514.1)
Supplement: Peer review history

# Reviewer report: Reviewer response for version 1

**DOI:** 10.21956/wellcomeopenres.21616.r92244

**Published:** 2024-08-12

**Authors:** Maurijn van der Zee

**Affiliations:** 1 Institute of Biology, Leiden University, Leiden, The Netherlands

Boyes and Boyes present here the genome sequence of *Anarisa innoxiella*. It is a high quality genome, sequenced with PacBio 78x coverage. Particularly the use of Hi-C data to scaffold the contigs into chromosomes make this assembly stand out from what was previously standard in the field of genome sequencing.

Are sufficient details of methods and materials provided to allow replication by others?

Yes

Is the rationale for creating the dataset(s) clearly described?

Yes

Are the datasets clearly presented in a useable and accessible format?

Yes

Are the protocols appropriate and is the work technically sound?

Yes

Reviewer Expertise:

comparative genomics, evo-devo, life histories

I confirm that I have read this submission and believe that I have an appropriate level of expertise to confirm that it is of an acceptable scientific standard.

# Reviewer report: Reviewer response for version 1

**DOI:** 10.21956/wellcomeopenres.21616.r92243

**Published:** 2024-08-12

**Authors:** Kay Lucek

**Affiliations:** 1 University of Neuchâtel, Neuchâtel, Switzerland

The authors present the chromosome level genome assembly of a female specimen of the Acer Sober, *Anarsia innoxiella*. The assembly consists of 31 chromosomes and is not annotated for genes. The assembly is highly complete as revealed by the high BUSCO score but not fully phased. Sequencing and genome assembly follow the current state of the art and use established methods. The assembly has been generated using the established pipeline of the Darwin Tree of Life Consortium.

 Overall, the presented assembly will be of great value to study cryptic diversification in moths, given that this species has only recently been split from another species based on mitochondrial barcode sequences. Once available, it would be interesting to perform a comparative analysis between the two species and contrast former taxonomical inferences.

Are sufficient details of methods and materials provided to allow replication by others?

Yes

Is the rationale for creating the dataset(s) clearly described?

Yes

Are the datasets clearly presented in a useable and accessible format?

Yes

Are the protocols appropriate and is the work technically sound?

Yes

Reviewer Expertise:

Speciation genomics

I confirm that I have read this submission and believe that I have an appropriate level of expertise to confirm that it is of an acceptable scientific standard.
